# Exploring the Platelet-to-Lymphocyte Ratio for Risk Stratification in Heart Failure: A Systematic Review

**DOI:** 10.7759/cureus.89522

**Published:** 2025-08-06

**Authors:** Mohamed Babikir Elbashir Hamid, Moayad H Ali, Abdalla Bashier Abdalla Elbashier, Lugien Ahmed Mohamed Ibrahim, Mustafa Eisa Ahmed Elhaj, Fatima Babikir Ali Mohamed, Aalia Omer Yousif Mohamedahmed, Abdalrahman Fathalrahman Abdalrahman Abdalmagid

**Affiliations:** 1 Cardiology, Prince Mohammad Bin Abdulaziz Hospital, Al-Madinah, SAU; 2 Cardiology, Hamad Medical Corporation, Doha, QAT; 3 General Practice, Urgent Care Center, Sakaka, SAU; 4 General Practice, Institute Of Endemic Diseases, Khartoum, SDN; 5 Cardiology, James Cook University Hospital, Middlesbrough, GBR; 6 Internal Medicine, University of Bahri, Alkadroo, SDN; 7 Internal Medicine, Sudan Medical Council, Khartoum, SDN; 8 Internal Medicine, Jaber Al-Ahmad Hospital, Al Jahra, KWT

**Keywords:** heart failure, inflammation, platelet-to-lymphocyte ratio, prognosis, risk stratification, systematic review

## Abstract

Heart failure (HF) remains a global health challenge with high morbidity and mortality, necessitating reliable biomarkers for risk stratification. The platelet-to-lymphocyte ratio (PLR), an emerging inflammatory marker, has shown prognostic potential in cardiovascular diseases, but its utility in HF remains inconsistently reported. This systematic review synthesizes evidence on PLR’s prognostic value in HF, focusing on mortality, hospitalization, and its role in multimarker models. We searched four databases -PubMed, Scopus, Web of Science, and Cochrane Library - for English-language observational studies published between January 2020 and June 2025, following Preferred Reporting Items for Systematic Reviews and Meta-Analyses (PRISMA) guidelines. Fourteen studies (n=14) were included after screening 172 records. Inclusion criteria comprised adult HF patients with PLR assessed as a prognostic factor; exclusions included reviews, editorials, abstracts without full data, animal studies, and non-English publications. Data on study characteristics, PLR cut-offs, outcomes, effect estimates, and adjustment covariates were extracted. Risk of bias was assessed using the Newcastle-Ottawa Scale. A meta-analysis was not performed due to high heterogeneity in study design, PLR measurement methods, and outcome definitions. Heterogeneity was further evaluated narratively based on methodological inconsistencies, differences in population characteristics, and statistical adjustments. Elevated PLR was significantly associated with increased mortality in ICU and acute HF settings, particularly when combined with the neutrophil-to-lymphocyte ratio (NLR), suggesting additive prognostic value in multimarker models. In contrast, PLR showed limited predictive utility in stable or community-dwelling HF cohorts. Risk of bias findings influenced interpretation, with stronger associations observed in studies with low bias scores. PLR cut-off thresholds varied substantially across studies, affecting comparability. While PLR adds incremental value in acute settings, especially when integrated with other inflammatory markers, its standalone use in chronic HF remains uncertain. Standardization of PLR measurement and further prospective research are essential to clarify its pathophysiological role and clinical applicability.

## Introduction and background

Heart failure (HF) remains a global public health challenge, affecting over 64 million individuals worldwide and imposing a significant burden on healthcare systems due to its high morbidity, mortality, and frequent hospitalizations [1]. Despite advancements in pharmacological and device-based therapies, the prognosis of HF patients remains poor, with a five-year mortality rate comparable to that of many malignancies [2]. This underscores the critical need for simple, reliable, and cost-effective biomarkers that can aid in early risk stratification, prognostication, and individualized management of HF [3].

Emerging evidence suggests that inflammation plays a central role in the pathophysiology of HF, contributing to myocardial remodeling, fibrosis, and progressive functional decline [4]. Among various hematologic inflammatory markers, the platelet-to-lymphocyte ratio (PLR), calculated from routine complete blood counts, has gained attention as a potential prognostic indicator [5]. PLR reflects the balance between thrombotic activation and immune competence: platelets, as mediators of thrombosis and inflammation, are often elevated in cardiovascular conditions [6], whereas lymphopenia serves as a marker of systemic stress and adverse outcomes [7]. PLR has been explored across a range of diseases, including acute coronary syndromes, stroke, and malignancies [8], and more recently in both acute and chronic HF cohorts across ICU, inpatient, and community settings [9,10].

However, the PLR's role in HF remains incompletely elucidated, particularly in comparison to established biomarkers such as N-terminal pro-B-type natriuretic peptide (NT-proBNP), which remains the standard for HF diagnosis and prognostication. Unlike NT-proBNP, which directly reflects myocardial stress, PLR may capture distinct inflammatory and thrombotic pathways [4]. Several retrospective and prospective studies have examined the prognostic value of PLR in HF, reporting variable associations with outcomes such as all-cause mortality, cardiovascular mortality, and risk of readmission [9,10]. Heterogeneity in the findings may be attributable to differences in study design, HF phenotype (acute vs. chronic), population characteristics, PLR cut-offs, and statistical adjustments.

Although previous narrative reviews and meta-analyses have investigated PLR in HF [9,10], many focused narrowly on short-term outcomes or included older studies with outdated HF classifications. This review seeks to update and expand upon existing literature by incorporating more recent studies, from 2020 to 2025, and evaluating the prognostic value of PLR across diverse HF phenotypes. Importantly, we aim to assess whether PLR adds incremental prognostic value beyond clinical models and established biomarkers, and how it performs within multimarker frameworks compared to other indices such as neutrophil-to-lymphocyte ratio (NLR) or monocyte-to-lymphocyte ratio (MLR). The rationale for focusing on PLR stems from its dual inflammatory-thrombotic relevance, ease of measurement, and emerging integration into risk stratification models.

Given this background, this systematic review aims to comprehensively evaluate and synthesize the current evidence on the prognostic significance of PLR in patients with HF. Specifically, it examines whether PLR is independently associated with adverse outcomes, including mortality, hospitalization, and clinical worsening, and explores its potential utility as an accessible, cost-effective tool for clinical decision-making. By consolidating the available literature, this review intends to inform future research directions and contribute to optimizing prognostic assessment in HF management.

## Review

Methods

Review Protocol

This systematic review was conducted in accordance with the Preferred Reporting Items for Systematic Reviews and Meta-Analyses (PRISMA) guidelines to ensure methodological rigor and transparency [11]. The review protocol was not registered in International Prospective Register of Systematic Reviews (PROSPERO) or any other registry due to time constraints.

Eligibility Criteria

Studies were included if they evaluated the prognostic value of PLR in adult patients (≥18 years) with a clinical diagnosis of HF. Eligible study designs were observational, including prospective or retrospective cohort studies and case-control studies, published in peer-reviewed journals between January 2020 and June 2025. This time frame was selected to reflect contemporary clinical practice and recent advancements in HF diagnosis and management. We excluded reviews, editorials, letters, case reports, conference abstracts without full data, animal studies, grey literature (e.g., theses, dissertations, trial registries), and studies not published in English.

Information Sources

A comprehensive literature search was conducted in four major databases: PubMed, Scopus, Web of Science, and the Cochrane Library. The last search was performed on 15 June 2025. We also manually screened reference lists of included studies and relevant reviews to identify additional eligible studies.

Search Strategy

The search strategy combined Medical Subject Headings (MeSH) and keywords related to "platelet-to-lymphocyte ratio" and "heart failure" using Boolean operators. The search strategy was tailored to each database and is detailed in Table 1.

**Table 1:** Search strategy for all databases

Database	Search strategy
PubMed	(“platelet to lymphocyte ratio” OR PLR) AND (“heart failure” OR “cardiac failure” OR “congestive heart failure”)
Scopus	TITLE-ABS-KEY (“platelet to lymphocyte ratio” OR PLR) AND TITLE-ABS-KEY (“heart failure” OR “cardiac failure” OR “congestive heart failure”) AND PUBYEAR > 2019
Web of Science	TS=(“platelet to lymphocyte ratio” OR PLR) AND TS=(“heart failure” OR “cardiac failure” OR “congestive heart failure”) AND PY=2020-2025
Cochrane Library	(“platelet to lymphocyte ratio” OR PLR) in Title Abstract Keyword AND (“heart failure” OR “cardiac failure” OR “congestive heart failure”) in Title Abstract Keyword, Publication Year from 2020 to 2025

Study Selection

All records were imported into EndNote (Clarivate, London, UK) for de-duplication. Two independent reviewers screened titles and abstracts for eligibility, followed by full-text screening. Disagreements were resolved by discussion or consultation with a third reviewer. Inter-reviewer agreement was assessed using Cohen’s kappa statistic to ensure consistency during screening.

Data Extraction

Two reviewers independently extracted data using a standardized data extraction form. Extracted data included author, year, country, study design, sample size, HF phenotype (acute, chronic, heart failure with reduced ejection fraction (HFrEF), heart failure with preserved ejection fraction (HFpEF)), diagnostic criteria, PLR measurement details (timing, calculation, cut-offs), outcomes assessed (e.g., mortality, rehospitalization, worsening HF), adjusted variables, statistical methods, and effect estimates. Where necessary, authors were contacted for clarification or missing data.

Risk of Bias Assessment

Risk of bias was assessed using the Newcastle-Ottawa Scale (NOS) [12], evaluating selection, comparability, and outcome domains. Two reviewers conducted the assessments independently, with discrepancies resolved through consensus. Inter-reviewer agreement for bias assessment was also quantified using the kappa statistic. Study quality (i.e., NOS score) did not determine inclusion but was considered during interpretation, with sensitivity inferences drawn more cautiously for studies with moderate NOS scores.

Data Synthesis

Meta-analysis was not performed due to heterogeneity in HF phenotypes, PLR measurement timing, cut-off values, outcome definitions, and statistical methods. Instead, a structured narrative synthesis was undertaken. This synthesis was organized by clinical setting (e.g., ICU vs. community), HF phenotype (e.g., acute HF, chronic HF, HFrEF, HFpEF), and outcomes (e.g., mortality, hospitalization). Differences in prognostic value across subgroups and study designs were described narratively. Subgroup analyses were not statistically conducted due to inconsistent reporting and limited data granularity across included studies.

Reporting Bias Assessment

Given the qualitative synthesis approach, statistical assessment of publication bias (e.g., funnel plots) was not applicable. Nonetheless, comprehensive database coverage, dual independent screening, and inclusion of recent studies aimed to reduce the risk of reporting bias.

Results

Selection Process

**Figure 1:**
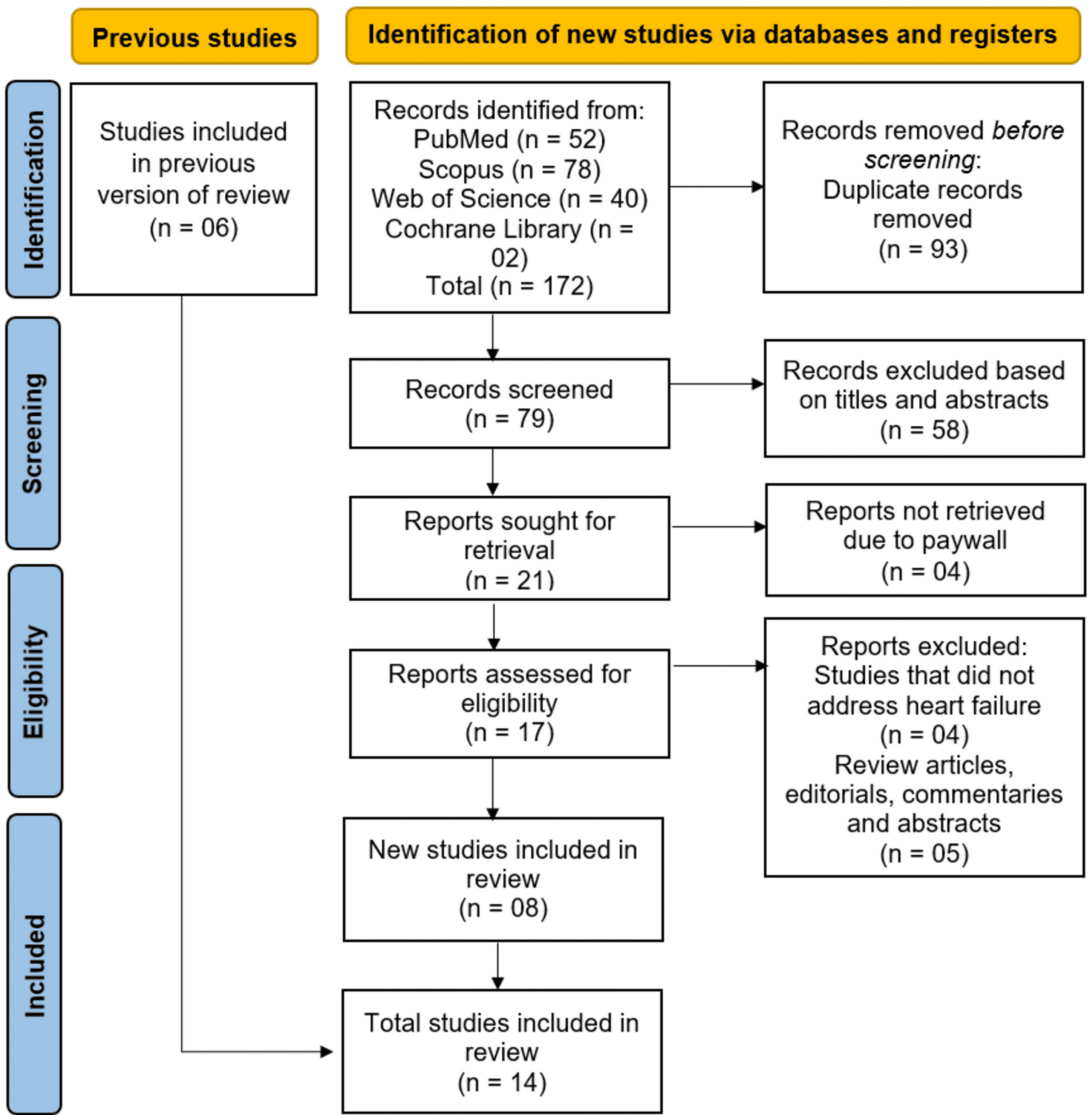
Studies selection process illustrated as a PRISMA flowchart PRISMA: Preferred Reporting Items for Systematic reviews and Meta-Analyses

The study selection followed the PRISMA guidelines and is illustrated in Figure 1.

We identified 172 records through systematic searches of PubMed (n=52), Scopus (n=78), Web of Science (n=40), and the Cochrane Library (n=2). After removing 93 duplicates, 79 studies underwent title and abstract screening, of which 58 were excluded. Full texts of 21 potentially eligible articles were sought, but four could not be accessed due to paywall restrictions. We attempted access through institutional databases and searched for open-access versions; however, full texts were unavailable and authors were unresponsive, leading to their exclusion. Of the remaining 17, nine were excluded (four not related to HF and five were reviews or editorials). Fourteen studies [13-26] were ultimately included (Figure 1). Six were carried forward from a previous review; duplication was avoided by re-evaluating for overlapping data. Among studies using the Medical Information Mart for Intensive Care (MIMIC)-IV database [13,15], patient cohorts and timeframes were compared to confirm non-overlap.

The included studies comprised retrospective and prospective cohort designs, along with two descriptive studies [20,24] which were retained for their unique contexts despite lower methodological rigor, with limitations considered during interpretation. PLR cut-off values varied and were not standardized across studies; thresholds included tertiles, quartiles, and empirical values (e.g., >193 or >75th percentile). Similarly, the timing of PLR measurement differed (e.g., at admission, during hospitalization), and this variability was acknowledged during synthesis. The diagnosis criteria for HF also varied but were extracted and reported per study to reflect any inconsistencies. Trends indicated that elevated PLR was more consistently associated with increased mortality in acute and ICU settings, while associations with rehospitalization or worsening HF were mixed. Some studies reported multivariable-adjusted outcomes, while others provided unadjusted estimates; these differences were clearly noted and considered in evaluating the overall evidence.

Study Characteristics

This systematic review included 14 studies [13-26] published between 2020 and 2025, conducted across diverse geographical regions including the USA, Romania, Japan, Turkey, China, Iran, Germany, and Austria. Study designs varied, with retrospective cohort studies being the most common (n=9) [13-15,18-21,23,26], followed by prospective cohort studies (n=3) [17,22,25], and descriptive or observational studies (n=2) [16,24]. Sample sizes ranged from 101 to 7,217 participants. Study populations included critically ill ICU patients [13,15], community-based individuals [16], and hospitalized patients with HFrEF or HFpEF [17,18,21,26].

It is important to note that a significant portion of the included studies focused on acutely ill or hospitalized populations, limiting generalizability to outpatient or early-stage HF settings. For instance, Hu et al. [13] analyzed over 7,000 ICU patients with HF, representing a severely ill cohort in whom PLR may reflect late-stage disease processes rather than serve as an early diagnostic or screening tool. Only a few studies, such as Wu et al. [16], assessed PLR in broader, community-based populations, highlighting the need for more research in ambulatory or early-diagnosis settings. Follow-up durations varied widely, from in-hospital outcomes to 5.5 years (median 66 months in Wu et al. [16]). A detailed summary of study characteristics is provided in Table 2.

**Table 2:** Characteristics of the included studies SOFA: Sequential Organ Failure Assessment; APS III: Acute Physiology Score III; MIMIC-IV: Medical Information Mart for Intensive Care-IV; CHF: Chronic Heart Failure; NR: Not reported; PLR: Platelet-to-lymphocyte ratio; LOS: Length of stay; NT-proBNP: N-terminal pro-B-type natriuretic peptide; RHD: Rheumatic Heart Disease; NHANES: National Health and Nutrition Examination Survey; AUC: Area under curve; NPAR: Neutrophil Percentage-to-Albumin Ratio; MLR: Monocyte-to-Lymphocyte Ratio; NLR: Neutrophil-to-Lymphocyte Ratio; HFpEF: Heart failure with preserved ejection fraction; LVEF: Left Ventricular Ejection Fraction; ICD: Implantable Cardioverter-Defibrillator; m-HALP: modified Hemoglobin, Albumin, Lymphocyte, and Platelet; HFrEF: Heart failure with reduced ejection fraction; PNI: Prognostic Nutritional Index; PROVE/HF: Persian Registry Of cardioVascular diseasE/Heart Failure; ADHF: Acute Decompensated Heart Failure; CVD: Cardiovascular Disease; Cr: Creatinine; COPD: Chronic Obstructive Pulmonary Disease; BP: Blood pressure; NYHA: New York Heart Association

Author (Year)	Country/Setting	Study design	Sample size (n)	Population characteristics	Heart failure definition/ Diagnosis criteria	PLR measurement details	PLR cut-off value (if used)	Follow-up duration	Primary outcomes reported	Key findings
Hu et al. (2014) [13]	USA/ICU patients from MIMIC-IV database	Retrospective cohort study	7,217	Critically ill ICU patients with heart failure	NR	PLR measured and stratified into tertiles (0–126.45, 126.45–252.40, 252.40–1000)	Tertile ranges used; no single cut-off reported	1 year	All-cause mortality	Higher PLR predicted greater 1-year mortality (36% increase in top tertile; 17% per tertile rise), especially in hypertensives; adding PLR improved SOFA and APS III models.
Cristescu et al. (2024) [14]	Romania	Retrospective analysis	427 CHF admissions	Mean age 68.48 ± 11.53 years; males 57.84%	NR	PLR measured; mean PLR = 144.84 ± 83.08	Not used	90-day readmission	LOS, 90-day readmission rates, correlation with NT-proBNP (in subcohort)	PLR did not show significant association with LOS; low positive correlation with NT-proBNP (r=0.151, p=0.006) in subanalysis.
Zhang and Ni (2025) [15]	USA/MIMIC-IV database (ICU setting)	Retrospective cohort study	1002	Critically ill patients with RHD admitted to ICU	RHD patients identified from MIMIC-IV database	PLR calculated from platelet and lymphocyte counts; divided into tertiles for analysis	Tertiles	30 days	30-day all-cause mortality	Elevated PLR was an independent predictor of increased 30-day mortality (adjusted HR 2.53, 95% CI: 1.87–3.42, p<0.001); risk increased progressively with higher PLR levels.
Wu et al. (2023) [16]	USA/NHANES database 2005–2016	Longitudinal cohort study	1,207	Adults aged ≥20 years with heart failure, community-dwelling individuals	Identified from NHANES HF data	PLR measured	Quartiles used; specific cut-off values not provided	Median 66.0 months	All-cause mortality	Elevated PLR was not associated with mortality risk (AUC = 0.58). NPAR and NLR were significantly associated with increased mortality, but PLR showed low predictive performance
Tamaki et al. (2023) [17]	Japan (Multicenter)	Prospective Multicenter Observational Study	1026	Patients with HFpEF admitted for acute decompensated heart failure	HFpEF; definition criteria not specified in abstract but participants were from a dedicated HFpEF registry	PLR measured at admission	>193 (optimal cut-off for predicting cardiac death)	Median 429 days	Cardiac death	High PLR (>193) independently associated with cardiac death; combination of high NLR and PLR strongest predictor (HR 2.66, 95% CI 1.51–4.70, p=0.0008)
Çakır (2023)[18]	Türkiye/Single center outpatient clinic	Cohort	180	HF patients, LVEF ≤35%, ICD for primary prevention	LVEF <35% by echo, ICD indicated	Blood PLR measured	NR	1 year	Cardiac mortality	NLR predicted 1-year mortality; PLR results NR
Liu et al., (2022) [19]	China/First Affiliated Hospital of Chongqing Medical University	Retrospective cohort study	550	Patients with CHF admitted from Jan 2018 to Apr 2020	CHF	PLR measured as systemic inflammatory marker	NR	June 2018 – May 31, 2022	Cardiovascular readmission and all-cause death	PLR was an independent prognostic factor for adverse outcomes; included in nomogram with traditional factors to predict mortality and readmission with good calibration and C-index values.
Kocaoglu and Alatli (2022) [20]	Turkey/Emergency Department of Balikesir University Hospital	Descriptive study	101	Mean age 73.15±10.19 years; 51.5% females, 48.5% males; patients diagnosed with acute heart failure	Diagnosis of acute heart failure	PLR measured at presentation; exact assay method not specified	NR	1-week and 3-month follow-up	1-week and 3-month mortality	PLR values were compared between survivors and non-survivors, but specific association or cut-off for PLR not reported; platelet count and NLR were significant predictors of mortality; m-HALP score was a good prognostic index for 3-month mortality.
Davran et al. (2023) [21]	Turkey/Coronary Intensive Care Unit	Observational	139	Patients admitted with decompensated HFrEF; EF <40%; Mean age ~69 years; both genders	Heart failure with reduced ejection fraction (EF <40%) decompensated for any reason	PLR measured; details on timing or lab method not specified	NR	1 year	Mortality (in-hospital or within 1-year follow-up)	PLR significantly higher in deceased group; Low PNI associated with worse prognosis and higher PLR; PLR linked to mortality and inflammation
Heidarpour et al. (2021) [22]	Iran/PROVE/HF registry, emergency department	Retrospective cohort study	405	Patients with acute decompensated heart failure; mean age 65.9 ± 13.49 years; 67.7% male	ADHF diagnosed at hospital admission	PLR calculated as platelet count divided by absolute lymphocyte count; categorized into quartiles	Quartiles used; specific cut-off values not reported	Mean 4.26 ± 2.2 months	CVD mortality, creatinine rise, rehospitalization	PLR was not significantly associated with CVD mortality or rehospitalization; minimal reduction in mortality in 2nd vs 1st quartile; Cr rise showed no significant association.
Delcea et al. (2021) [23]	Romania/Cardiology Department, tertiary hospital	Retrospective observational study	1299	Mean age 72.35 ± 10.45 years; 51.96% women; hospitalized HF patients	HF diagnosis based on admission to cardiology department	PLR measured from blood samples at admission	NR	In-hospital (during hospitalization)	In-hospital all-cause mortality; extended length of stay (>7 days)	PLR was an independent predictor of extended length of stay but not an independent predictor of in-hospital mortality when adjusted for NT-proBNP, dyspnea at rest, COPD, age, and systolic BP; MLR was the strongest predictor of mortality.
Dahlen et al. (2021) [24]	Germany/MyoVasc study	Observational cohort study	3250	Subjects enrolled in the MyoVasc study investigating cardiac function and heart failure phenotypes	NR	PLR calculated from blood samples	>75th percentile used as cut-off for PLR	NR	Worsening of HF	PLR >75th percentile was associated with increased risk of worsening heart failure, independent of potential confounders
Arfsten et al. (2021) [25]	Austria	Prospective cohort study	443 HFrEF patients (total 818 including 375 cancer patients)	Stable HFrEF patients	NR (HFrEF diagnosis likely based on reduced ejection fraction)	PLR calculated at index day	NR	NR	Association of PLR with disease severity (NT-proBNP, NYHA class) and all-cause mortality	PLR was significantly associated with disease severity and all-cause mortality; discriminatory power confirmed by Kaplan-Meier analysis; risk per PLR increment was higher in HFrEF compared to cancer patients
Sadeghi et al. (2020) [26]	Iran/Tertiary referral center	Retrospective study	197	Patients with HFrEF admitted between Jan 2016 – Jan 2017	HFrEF diagnosis	PLR measured from blood counts	NR	6 months	Short-term mortality	Higher PLR was associated with mortality in univariate analysis (P=0.006), but PLR was not an independent predictor of short-term mortality in multivariate Cox analysis

Association of PLR with Mortality Outcomes

Elevated PLR was significantly associated with increased mortality risk in multiple studies, though the strength and consistency of this association varied based on population, follow-up duration, and analytical approach. Most studies assessed all-cause mortality [13,15,16,21,25,26], while a few specifically evaluated cardiovascular mortality [17,22]. In ICU settings, Hu et al. [13] reported that the highest PLR tertile (252.40-1000) was independently associated with a 36% higher one-year all-cause mortality risk compared to the lowest tertile (adjusted HR 1.36, 95% CI: 1.23-1.50, p<0.001), with each tertile increment corresponding to a 17% increase in risk. Similarly, Zhang and Ni [15] found PLR to be a strong predictor of 30-day all-cause mortality in critically ill patients with rheumatic heart disease (RHD; adjusted HR 2.53, 95% CI: 1.87-3.42, p<0.001), with a dose-response relationship observed. Tamaki et al. [17] demonstrated that a PLR >193, especially when combined with high neutrophil-to-lymphocyte ratio (NLR; >4.5), was associated with a 2.66-fold higher risk of cardiac death in acute HFpEF (95% CI: 1.51-4.70, p=0.0008), highlighting the utility of multimarker models.

However, inconsistencies emerged in longer-term and community-based settings. Wu et al. [16] found no significant association between PLR and long-term all-cause mortality in community-dwelling HF patients (AUC = 0.58, 95% CI: 0.55-0.61), suggesting limited prognostic value over extended follow-up. Heidarpour et al. [22] also observed no significant link between PLR quartiles and cardiovascular mortality (HR 0.40, 95% CI: 0.16-1.01, p=0.054), while Sadeghi et al. [26] reported a significant univariate association that lost significance after multivariate adjustment. Notably, the studies varied in their adjustment strategies. Some controlled for key confounders such as age, sex, comorbidities, NT-proBNP, and ejection fraction [13,15,17,19,23], while others either provided unadjusted estimates or adjusted for fewer variables [14,16,22,26], limiting comparability.

There was substantial heterogeneity in PLR cut-offs across studies, ranging from data-driven tertiles and quartiles [13,15,22] to specific thresholds (e.g., >193 in Tamaki et al. [17]; >75th percentile in Dahlen et al. [24]), which likely influenced the strength and direction of the associations. Additionally, follow-up durations varied widely, from one week [20] to 5.5 years [16], and PLR appeared more predictive of short-term outcomes in acutely ill or ICU settings than in long-term community cohorts. This may reflect the acute inflammatory and thrombotic burden in hospitalized patients, where PLR serves as a surrogate for disease severity and systemic stress.

Importantly, several studies assessed whether PLR added value to existing clinical models or biomarkers. Hu et al. [13] demonstrated that incorporating PLR improved predictive performance of the Sequential Organ Failure Assessment (SOFA) and Acute Physiology Score (APS) III scores, while Liu et al. [19] included PLR in a nomogram alongside New York Heart Association (NYHA) class and creatinine, showing improved calibration and discrimination (C-index=0.78). However, thresholds for clinical utility, such as a specific improvement in area under curve (AUC) or net reclassification index, were not consistently reported, and no studies validated the findings externally in independent cohorts. Similarly, sensitivity analyses were rarely performed, limiting the generalizability of results. Collectively, these findings suggest that PLR may be most useful as part of multimarker models in acute care settings but is less reliable as a standalone prognostic tool in stable, outpatient populations.

PLR and Other Clinical Outcomes

Beyond mortality, PLR was evaluated for its association with hospital length of stay (LOS), readmission, and worsening HF. Cristescu et al. [14] reported no significant association between PLR and LOS in chronic HF patients but noted a weak positive correlation with NT-proBNP levels (r=0.151, p=0.006). Delcea et al. [23] identified PLR as an independent predictor of extended LOS (greater than seven days) in hospitalized HF patients (p<0.05), though its predictive value was weaker than MLR. Dahlen et al. [24] found that PLR >75th percentile was associated with a 50% increased risk of worsening HF (HR 1.50, 95% CI: 1.17-1.93), independent of confounders.

PLR as a Part of Multimarker Models

Several studies incorporated PLR into multimarker models or nomograms to improve risk stratification. Liu et al. [19] developed a nomogram integrating PLR with traditional prognostic factors (e.g., NYHA class, creatinine) to predict adverse outcomes in chronic HF, demonstrating good calibration (C-index=0.78). Similarly, Tamaki et al. [17] highlighted the superior predictive power of combining PLR and NLR for cardiac death in HFpEF. Conversely, Kocaoglu and Alatli [20] reported that PLR alone lacked prognostic significance for one-week or three-month mortality in acute HF, though platelet count and NLR were predictive.

Heterogeneity in PLR Cut-offs and Measurement

The included studies showed substantial heterogeneity in how PLR was measured, categorized, and analyzed, which affected the consistency and comparability of findings. Most studies measured PLR at hospital admission [13-15,17-19,21,22,23,26], while a few did not clearly specify the timing [20,24,25], and none used discharge or follow-up values. Cut-off definitions varied widely: some used data-driven categories such as tertiles [13,15] or quartiles [16,22]; others applied empirically derived thresholds (e.g., >193 in Tamaki et al. [17], >75th percentile in Dahlen et al. [24]); and several treated PLR as a continuous variable without categorical thresholds [19,25]. These differences were not statistically harmonized, and no subgroup analyses were performed to explore the impact of categorization methods.

All studies calculated PLR from routine blood counts, but units and laboratory standards were not consistently reported, limiting standardization. Adjustment for confounders also varied: while some studies controlled for clinical and inflammatory variables (e.g., age, comorbidities, NT-proBNP, ejection fraction) [13,15,17,19,23], others reported only unadjusted associations [14,16,20,21,26], complicating interpretation of independent effects. A few studies did not clearly report effect estimates or statistical significance for PLR, despite inclusion in their models, most notably, Çakır [18] and Kocaoglu and Alatli [20].

Null associations were observed in several adjusted models. For example, Wu et al. [16] and Heidarpour et al. [22] found no significant link between PLR and long-term or cardiovascular mortality after adjustment, while Sadeghi et al. [26] reported a significant univariate association that disappeared in multivariate analysis. These findings suggest that PLR's prognostic value may be confounded or population-dependent. Sample sizes also varied widely, from as few as 101 [20] to over 7,000 participants [13], which likely influenced statistical power and precision of estimates across studies. Table 3 summarizes the differences in PLR measurement, effect estimates, and analytical approaches.

**Table 3:** Association of the platelet-to-lymphocyte ratio (PLR) with outcomes LOS: Length of stay; NT-proBNP: N-terminal pro-B-type natriuretic peptide; RHD: Rheumatic Heart Disease; NR: Not reported; AUC: Area under curve; BMI: Body Mass Index; NYHA: New York Heart Association; LMR: Lymphocyte-to-Monocyte Ratio; CHF: Chronic Heart Failure; ROC: Receiver operating characteristics; HFrEF: Heart Failure with Ejection Fraction; NS: Not Significant; OPTIMIZE-HF: Organized Program to Initiate Lifesaving Treatment in Hospitalized Patients with Heart Failure; COPD: Chronic Obstructive Pulmonary Disease; BP: Blood Pressure; MLR: Monocyte-to-Lymphocyte Ratio; HF: Heart Failure.

Author (Year)	PLR category/Analysis method	Adjusted covariates	Outcome assessed	Effect estimate (95% CI)	p-value	Interpretation
Hu et al. (2024) [13]	Tertiles (0–126.45, 126.45–252.40, 252.40–1000)/Cox proportional hazards models	Adjusted	1-year all-cause mortality	HR 1.36 (95% CI: 1.23–1.50) for highest vs. lowest tertile	<0.001	Elevated PLR was independently associated with higher 1-year mortality; highest tertile had 36% increased risk compared to lowest tertile. Each tertile increment corresponded to a 17% rise in risk.
Cristescu et al. (2024) [14]	PLR (continuous)/Pearson correlation	None (unadjusted)	LOS (no significant association), NT-proBNP (r=0.151)	r=0.151 (NT-proBNP)	p=0.006 (NT-proBNP), p>0.05 (LOS)	PLR was not associated with LOS but showed a weak positive correlation with NT-proBNP.
Zhang and Ni (2025) [15]	Tertiles (elevated PLR vs. lower)/Multivariate Cox proportional hazards analysis with restricted cubic spline analysis	Potential confounders	30-day all-cause mortality	Adjusted HR: 2.53 (95% CI: 1.87–3.42)	<0.001	Elevated PLR was independently associated with increased risk of 30-day all-cause mortality in critically ill RHD patients; risk progressively increases with higher PLR levels.
Wu et al. (2023) [16]	Highest vs. lowest quartile/Multivariable Cox regression	NR	All-cause mortality	Not significant (AUC=0.58, 95% CI: 0.55–0.61)	NR	Elevated PLR was not associated with mortality risk
Tamaki et al. (2023) [17]	High PLR (>193) alone and in combination with high NLR (>4.5); Cox regression analysis	NR	Cardiac death	HR 2.66 (95% CI: 1.51–4.70) for both high NLR and PLR combined	0.0008	High PLR, especially when combined with high NLR, is independently associated with increased risk of cardiac death in acute HFpEF patients
Çakır (2023)[18]	NR	NR	One-year cardiovascular mortality	NLR: OR 1.328 (95% CI: 1.129–1.563)	p < 0.01	Higher NLR independently associated with increased 1-year cardiovascular mortality; PLR results not reported
Liu et al. (2022) [19]	Continuous (Cox multivariate regression)	Age, BMI, NYHA classification, creatinine, LMR, PLR	Adverse outcomes (cardiovascular readmission and all-cause death)	NR	0.015	PLR was an independent prognostic factor; higher PLR associated with increased risk of adverse outcomes in CHF patients
Kocaoglu and Alatli (2022) [20]	PLR (ROC analysis)	NR	1-week and 3-month mortality	NR	Platelet p=0.018 (1-week), p=0.006 (3-month)	PLR analysed but specific results not reported; platelet count higher in survivors; no effect estimate or significance reported for PLR
Davran et al. (2023) [21]	Higher PLR vs. lower PLR (group comparison)	NR	1-year mortality	NR	Significant	Patients who died within 1 year had significantly higher PLR, suggesting elevated PLR is associated with increased mortality in HFrEF.
Heidarpour et al. (2021) [22]	PLR Quartiles (2nd vs 1st); Multivariate Cox model	NR	CVD mortality, creatinine rise	HR 0.40 (0.16–1.01) for mortality; not reported for creatinine rise	0.054 for mortality; NS for creatinine rise	No significant association with mortality (trend towards reduced risk in 2nd quartile) or creatinine rise
Delcea et al. (2021) [23]	PLR (Cox regression & multivariable logistic regression)	OPTIMIZE-HF model parameters; NT-proBNP, dyspnea at rest, COPD, age, systolic BP (for mortality); covariates not detailed (for LOS)	In-hospital mortality; Extended length of stay (>7 days)	Reported as independent predictor	<0.05 (both outcomes)	PLR was an independent predictor of in-hospital mortality and extended LOS, though MLR showed stronger predictive value when adjusted for clinical parameters
Dahlen et al. (2021) [24]	PLR>75th percentile (Cox regression)	Potential confounders	Worsening of HF	HR = 1.50 (1.17 – 1.93)	NR	High PLR (>75th percentile) was associated with a 50% increased risk of worsening heart failure independent of confounders.
Arfsten et al. (2024) [25]	PLR (crude model, Kaplan–Meier, interaction analysis)	None (crude models)	All-cause mortality; risk increase per increment	NR	≤0.014 (mortality), log-rank ≤ 0.026, pinteraction = 0.005	PLR significantly associated with disease severity and all-cause mortality in HFrEF; stronger risk increase per increment compared to cancer patients.
Sadeghi et al. (2020) [26]	PLR/Cox multivariate analysis	NR	Six-month mortality in HFrEF patients	NR	0.006 (univariate), non-significant in multivariate	Although PLR was significantly higher in deceased patients on univariate analysis (p=0.006), it did not independently predict short-term mortality after adjustment

Summary of Key Findings

Overall, PLR demonstrated prognostic utility for mortality and adverse outcomes in specific HF populations, particularly in ICU and acute decompensated HF settings. However, its predictive value was inconsistent in community-based or stable HF cohorts, and its independent significance often depended on adjustment for confounders or integration with other biomarkers.

Results of Risk of Bias Assessment

Risk of bias was assessed using the original NOS, without adaptation. Two reviewers independently scored each study across the three NOS domains-selection, comparability, and outcome assessment-with disagreements resolved through discussion and consensus; inter-rater agreement was quantified using the kappa statistic. While NOS scores did not influence study inclusion, they were considered when interpreting results. Among the 14 included studies, seven were rated as low risk of bias (NOS score ≥7): Hu et al. [13], Zhang and Ni [15], Tamaki et al. [17], Liu et al. [19], Delcea et al. [23], Dahlen et al. [24], and Arfsten et al. [25]. These studies generally employed robust methodologies, had clearer outcome definitions, and adjusted for key confounders. However, even among these, divergent findings were noted. For example, Dahlen et al. [24] found only moderate associations, highlighting the complexity of PLR's prognostic role. The remaining seven studies were rated as moderate risk (NOS score 5-6), including Cristescu et al. [14], Wu et al. [16], Çakır [18], Kocaoglu and Alatli [20], Davran et al. [21], Heidarpour et al. [22], and Sadeghi et al. [26], with common limitations being insufficient adjustment for confounders, unclear exposure timing, or short follow-up. The comparability domain was most frequently underreported, posing a potential threat to internal validity. No studies were excluded due to the high risk of bias; this reflects the dataset itself, as none of the included studies scored below five. While subgroup analysis by NOS category was considered, it was not feasible due to limited statistical power and inconsistent outcome reporting. Finally, the NOS tool itself has inherent limitations in assessing retrospective studies and does not capture unmeasured confounding, warranting cautious interpretation of the associations reported. A full breakdown of the NOS scores by domain is presented in Table 4.

**Table 4:** Risk of bias assessment using the Newcastle-Ottawa Scale (NOS)

Study (Author, Year)	Selection (Max 4)	Comparability (Max 2)	Outcome (Max 3)	Total Score (Max 9)	Risk of bias
Hu et al. (2024) [13]	3	2	3	8	Low
Cristescu et al. (2024) [14]	3	1	2	6	Moderate
Zhang and Ni (2025) [15]	4	2	3	9	Low
Wu et al. (2023) [16]	3	1	2	6	Moderate
Tamaki et al. (2023) [17]	4	2	3	9	Low
Çakır (2023) [18]	2	1	2	5	Moderate
Liu et al. (2022) [19]	3	2	3	8	Low
Kocaoglu and Alatli (2022) [20]	2	1	2	5	Moderate
Davran et al. (2023) [21]	3	1	2	6	Moderate
Heidarpour et al. (2021) [22]	3	1	2	6	Moderate
Delcea et al. (2021) [23]	3	2	3	8	Low
Dahlen et al. (2021) [24]	4	2	3	9	Low
Arfsten et al. (2021) [25]	3	2	3	8	Low
Sadeghi et al. (2020) [26]	2	1	2	5	Moderate

Discussion

Prognostic Value of PLR in Heart Failure

The present systematic review evaluated the prognostic utility of PLR in HF across 14 studies, revealing a nuanced association between elevated PLR and adverse outcomes. In critically ill ICU patients with HF, higher PLR tertiles were independently linked to increased one-year mortality (adjusted HR 1.36, 95% CI: 1.23-1.50) [13] and 30-day mortality in RHD (adjusted HR 2.53, 95% CI: 1.87-3.42) [15]. These findings align with prior research emphasizing PLR as a marker of systemic inflammation and thrombotic activity, which exacerbates HF progression [27]. However, the association was less consistent in community-based cohorts (e.g., Wu et al. [16], AUC=0.58) and stable HFrEF populations [22,26], suggesting that PLR's predictive power may be context-dependent, with stronger utility in acute or decompensated HF. This aligns with PLR's reflection of acute-phase reactants (e.g., platelet activation during acute stress) rather than chronic inflammation, explaining its superior performance in critical care settings. This heterogeneity mirrors earlier meta-analyses, where PLR's prognostic significance varied by HF phenotype and clinical setting [28]. Notably, Tamaki et al. [17] demonstrated that combining PLR with NLR (>193 and >4.5, respectively) enhanced risk stratification for cardiac death in HFpEF (HR 2.66, 95% CI: 1.51-4.70), though this model awaits external validation.

PLR in Multimarker Models and Clinical Outcomes

Beyond mortality, PLR contributed to predictive models for hospital LOS, readmission, and HF worsening. Delcea et al. [23] identified PLR as an independent predictor of extended LOS (greater than seven days), though its effect was attenuated when adjusted for NT-proBNP and comorbidities. Similarly, Dahlen et al. [24] reported that PLR >75th percentile increased the risk of HF worsening by 50% (HR 1.50, 95% CI: 1.17-1.93), independent of traditional risk factors. However, definitions of "worsening HF" and "extended LOS" varied across studies, limiting cross-study comparability. These findings corroborate studies linking PLR to endothelial dysfunction and fluid retention, mechanisms that may prolong hospitalization [29]. Notably, PLR’s prognostic strength was influenced by the timing of measurement (e.g., admission vs. peak values), but insufficient data precluded subgroup analysis. The integration of PLR into nomograms, as in Liu et al. [19] (C-index=0.78), suggests its additive value when combined with clinical parameters like NYHA class and creatinine, though these models were derived from single cohorts without external validation. This aligns with emerging frameworks advocating for inflammation-based scores in HF management [30], but sex-specific or age-adjusted PLR thresholds were rarely explored, potentially overlooking demographic variations in inflammatory responses.

Heterogeneity in PLR Measurement and Cut-offs

A key challenge in interpreting the PLR's prognostic role is the lack of standardized measurement protocols and cut-offs. Studies employed tertiles [13,15], quartiles [16,22], or empirically derived thresholds (e.g., >193 [17]), with varying adjustment for confounders. Only six out of 14 studies [13,15,19,23-25] systematically adjusted for comorbidities (e.g., infection, anemia) or medications (e.g., corticosteroids) that may influence PLR. For instance, Hu et al. [13] and Zhang and Ni [15] controlled for severity scores (SOFA, APS III), whereas Wu et al. [16] and Heidarpour et al. [22] did not adjust for inflammatory comorbidities. This inconsistency may explain divergent findings, particularly in studies where PLR lost significance after multivariate adjustment [26]. Comparatively, NLR, a more widely studied ratio, has established cut-offs (e.g., >3.0) and stronger consensus in HF prognostication [31]. PLR’s underperformance relative to MLR or Systemic Immune-Inflammation Index (SII) [23,24] may stem from its weaker reflection of monocyte-driven inflammation, which is pivotal in chronic HF. The variability in PLR’s performance underscores the need for consensus-building efforts to standardize cut-offs and integrate PLR into existing HF risk scores (e.g., Meta-Analysis Global Group in Chronic Heart Failure or MAGGIC), alongside mechanistic studies to clarify its context-specific utility [32].

Biological Plausibility and Mechanistic Insights

PLR’s association with HF outcomes may reflect its dual role in inflammation and thrombosis. Elevated platelets (a numerator in PLR) correlate with hypercoagulability and microvascular thrombosis, exacerbating myocardial ischemia [33], while lymphopenia (denominator) signifies systemic inflammation and impaired adaptive immunity [34]. This acute-phase response dominates in decompensated HF, whereas chronic inflammation in stable HF may dilute PLR’s prognostic signal. For example, Arfsten et al. [25] reported that PLR’s discriminatory power for mortality was stronger in HFrEF than in cancer patients, suggesting disease-specific pathways. However, the weak correlation between PLR and NT-proBNP (r=0.151) observed by Cristescu et al. [14] implies that PLR may capture distinct pathophysiological processes, such as oxidative stress or platelet activation, rather than purely hemodynamic stress. The temporal stability of PLR in chronic HF remains unstudied, limiting insights into its reproducibility for longitudinal monitoring.

Limitations

This review has several limitations. First, the predominance of retrospective studies [13-15,18-21,23,26] introduces risks of selection bias and unmeasured confounding, as reflected in the moderate NOS scores of 7/14 studies [14,16,18,20-22,26]. Publication bias could not be assessed via funnel plots due to qualitative synthesis, and grey literature searches were not conducted. Second, heterogeneity in PLR measurement (timing, assay methods) and cut-offs precluded meta-analysis, limiting quantitative synthesis. Non-English studies meeting inclusion criteria were not identified, but their exclusion may have omitted relevant data. Third, most studies focused on short-term outcomes (e.g., 30-day mortality [15]), with limited data on PLR’s long-term predictive value. Finally, HF definitions and outcome measures (e.g., "worsening HF") lacked uniformity, reducing generalizability. These limitations underscore the need for prospective, multicenter studies with standardized PLR protocols and external validation of multimarker models.

## Conclusions

PLR shows promise as a prognostic biomarker in heart failure, particularly in acute and ICU settings, where elevated levels are consistently associated with increased short-term mortality and adverse outcomes such as extended hospitalization. Its value appears more evident in high-risk subgroups, including patients with HFpEF and those with heightened systemic inflammation, especially when used in combination with other markers like NLR. However, current evidence is insufficient to support PLR for routine clinical use, and it should still be considered investigational. Its predictive performance in stable or community-based HF remains inconsistent, with several studies showing null associations or loss of significance after multivariable adjustment.

These discrepancies highlight the need for context-specific interpretation and standardization of PLR measurement. Future research should focus on prospective validation in well-defined HF phenotypes (e.g., HFrEF vs. HFpEF, elderly vs. non-elderly), using harmonized biomarker panels and clinically relevant cut-off values. Although PLR is inexpensive and readily accessible, making it appealing for use in low-resource settings, there is limited evidence on its cost-effectiveness or practical impact on clinical decision-making. Currently, PLR may offer incremental value when added to established tools like NT-proBNP, but it does not independently drive treatment pathways. Therefore, its inclusion in future HF guidelines should await further confirmatory studies and consensus on standardized thresholds and clinical utility.
